# Angiotensin-Converting Enzyme Insertion/Deletion Polymorphism Contributes to Ischemic Stroke Risk: A Meta-Analysis of 50 Case-Control Studies

**DOI:** 10.1371/journal.pone.0046495

**Published:** 2012-10-01

**Authors:** Zhizhong Zhang, Gelin Xu, Dezhi Liu, Xinying Fan, Wusheng Zhu, Xinfeng Liu

**Affiliations:** Department of Neurology, Jinling Hospital, Nanjing University School of Medicine, Jiangsu Province, China; University of Hong Kong, China

## Abstract

**Background:**

Many studies have investigated the association between the *angiotensin-converting enzyme* (*ACE*) gene insertion/deletion (I/D) polymorphism and risk of ischemic stroke. However, the evidence is inadequate to draw robust conclusions because most studies were generally small and conducted in heterogeneous populations. To shed light on these inconclusive findings, we conducted a large meta-analysis of studies relating the *ACE* I/D polymorphism to the risk of ischemic stroke.

**Methods:**

Relevant studies were identified by searching PubMed and Embase through February 2012 and by reviewing the references of retrieved articles. We included studies that reported odds ratio (OR) with 95% confidence interval (CI) for the association between this polymorphism and ischemic stroke risk.

**Results:**

Fifty independent publications, with 10 070 stroke cases and 22 103 controls, were included. The results indicated that the DD homozygote carriers had a 37% higher risk of ischemic stroke when compared with the homozygotes II and heterozygote ID [odds ratio (OR) = 1.37, 95% confidence interval (CI): 1.22–1.53]. Subgroup analyses indicated that this higher risk was more pronounced among Asians, hospital-based studies, and small vessel disease (SVD). Potential publication bias may exist, but correction for this bias using a formal statistical method did not materially alter the combined risk estimate.

**Conclusion:**

The results of our meta-analysis indicate that the D allele of *ACE* I/D polymorphism is a low-penetrance susceptibility marker of ischemic stroke.

## Introduction

Stroke is a common neurological disease and a leading cause of death and long-term disability worldwide [1]. Strong evidence from genetic association studies indicates that genetic predisposition, in addition to such recognized risk factors as hypertension, smoking, diabetes, obesity, and advanced age, contributes to the development of stroke [2]. Identification and characterization of genetic variations that play such a role may allow improved prognostication, therapy, and prevention.

The renin-angiotensin system (RAS) is a hormonal signaling mechanism implicated in the atherosclerosis and regulation of blood pressure [3]. Angiotensin-converting enzyme (ACE), a key enzyme in the RAS, plays important roles in vascular remodeling, atherosclerosis, and ischemic stroke [4]–[6]. It catalyses the conversion of inactive angiotensin I to active angiotensin II, which is known to be involved in vascular hypertrophy, vasoconstriction, and atherosclerotic processes [7]. The human *ACE* gene is located on chromosome 17q23, where an insertion/deletion polymorphism (I/D, dbSNP rs4646994) in intron 16 has been identified [8]. This polymorphism is based on the presence (insertion, I) or absence (deletion, D) of a 287-bp DNA fragment. The D allele of this polymorphism has been associated with elevated serum ACE level in a codominant pattern and has been investigated as a potential susceptibility factor for ischemic stroke. A large number of studies have reported the association between the I/D polymorphism of *ACE* gene and the risk of ischemic stroke, but the results were inconclusive [9]–[12]. The association between this polymorphism with ischemic stroke risk has attracted widespread attention in recent years and has been a research focus. However, each of these studies typically contained a few subjects and therefore was neither adequate nor sufficiently informative to clearly demonstrate an association. Moreover, these studies varied markedly by including different populations, sampling strategies, genotyping procedures, and quality control. Previously published meta-analyses reported significant associations between *ACE* I/D and risk of ischemic stroke [13]–[19]. However, it remains unclear whether ethnicity, stroke subtype, subject source, and gender could affect the associations. Since then, additional many studies with a large sample size about this polymorphism on ischemic stroke risk have been reported. Subgroup analyses performed by ethnicity, stroke subtype, subject source, and gender were also possible now.

Therefore, we present herein the results of a large meta-analysis of published data investigating the association between *ACE* I/D and ischemic stroke for various genetic contrasts, in which we explored the between-studies heterogeneity and the existence of potential bias.

## Materials and Methods

### Literature Search and Selection

Two online electronic databases (PubMed and Embase) were searched for eligible articles through February 2012. The search was limited to English language full-text papers. Abstract, review or editorials were not included. The medical subject headings and terms used for the search were: stroke, brain infarction, and cerebrovascular disease in combination with ACE, angiotensin-converting enzyme, polymorphism, genotype, gene, or mutation. The references of all identified publications were searched for any additional studies, and the related articles option in PubMed was used to search for further potentially relevant articles.

Studies included in our meta-analysis have to meet the following criteria: (1) hypothesis-driven studies specific for *ACE* I/D polymorphism and provided cases of ischemic stroke (large-artery atherosclerosis, cardioembolic stroke, small-vessel stroke, or other determined and undetermined causes) and control subjects (population- or hospital-based controls), (2) had neuroimaging (CT or MRI) confirmation of an ischemic stroke diagnosis, (3) evaluation of *ACE* I/D polymorphism and ischemic stroke risk and (4) sufficient data for examining an odds ratio (OR) with 95% confidence interval (CI). Studies were excluded if: (1) patients were under 18 years of age, or (2) original genotype data was not reported. For duplicate publications, the smaller dataset was discarded.

### Data Extraction

Two investigators (Z.Z. and G.X.) independently extracted data and reached a consensus on all of the items. For each study, the following characteristics were collected: the first author's last name, year of publication, country of origin, ethnicity, matching conditions, numbers of genotyped cases and controls, the genotype distribution of cases and controls for the *ACE* I/D polymorphism, source of control groups (population- or hospital-based controls), and genotyping methods. Different ethnic descents were categorized as European, Asian and African.

### Statistical Analysis

For each study, we first examined whether the genotype distribution in controls was consistent with Hardy-Weinberg equilibrium (HWE) by χ^2^ test. To measure the strength of genetic association for *ACE* I/D polymorphism, the ORs, together with the 95% CIs were calculated. The statistical significance of the summary OR was determined with the Z test, and *P*<0.05 was considered as statistically significant. We first estimated the risks of the ID and DD genotypes on strokes, compared with the wild-type II homozygote, and then evaluated the risks of (ID/DD) vs II and DD vs (ID/II) on strokes, assuming dominant and recessive effects of the variant D allele, respectively. We also estimated the risks of D allele vs A allele. In addition, stratified analyses were performed by ethnicity, source of controls, subtype, gender, and HWE.

Heterogeneity among studies was assessed with the *Q*-test and *I* ^2^ statistics [20]. If there was no significant heterogeneity, the fixed-effects model (the Mantel-Haenszel method) was used to estimate the summary OR. Otherwise, the random-effects model (the DerSimonian and Laird method) was adopted. We conducted stratified analyses to explore possible explanations for heterogeneity and to test the robustness of the association.

Cumulative and recursive cumulative meta-analysis were performed to provide a framework for updating a genetic effect from all studies and to measure how much the genetic effect changes as evidence accumulates [21], [22]. Therefore, cumulative meta-analysis demonstrates the trend in risk effect, and recursive cumulative meta-analysis indicates the stability in risk effect.

We also performed sensitivity analyses to evaluate the stability of the results. A single study involved in the meta-analysis was deleted each time to reflect the influence of the individual data to the pooled ORs. For assessment of potential publication bias, we used funnel plots and Egger's linear regression test. Moreover, we performed the Duval and Tweedie nonparametric trim and fill procedure to further assess potential effects of publication bias [23]. This method considers the possibility of hypothetical missing studies, and recalculates a pooled estimate. All analyses were done with STATA version 11.0 (StataCorp, College Station, TX).

## Results

### Literature Search

**Figure 1:**
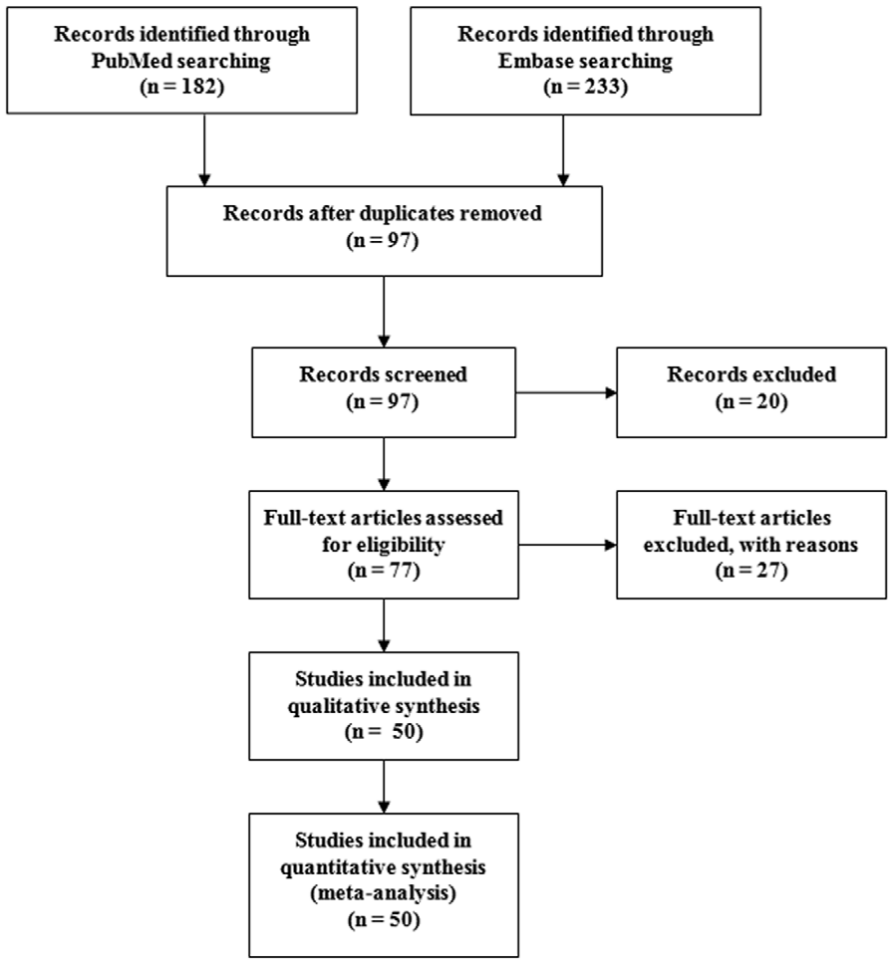
Flow diagram of the literature search.

A flow diagram of the literature search is shown in Figure 1. Total searches yielded 415 entries. Of these, 318 studies were excluded after reading the title or abstract because of obvious irrelevance to our study aim. Ninety-seven studies appeared to be potentially relevant for inclusion in our study. Twenty studies were excluded because of overlapping cases or their data were not extractable. Seventy-seven full-text articles were reviewed. Twenty-seven studies were further excluded for the following reasons: abstract, review or editorials (n = 22); no control population (n = 4); or children (n = 1). Therefore, a total of 50 studies met the inclusion criteria [9]–[12], [24]–[69].

### Study Characteristics

The characteristics of included studies are summarized in Table 1. The 50 included studies were published between 1994 and 2011 and comprised a total of 10 070 cases and 22 103 controls. There were 26 studies of Asian descendents, 23 studies of European descendents, and 1 study of African descendents. Of the 50 studies, 42 studies used frequency-matched controls to the cases by the age or sex. A classic polymerase chain reaction assay was performed in all of the 50 studies; however, only 20 (40%) studies mentioned quality control on genotyping. The genotype distributions among the controls of all studies were in agreement with HWE except for eight studies.

**Table 1:** Main characteristics of selected studies.

First author	Year	Country	Ethnicity	Sample size		Matching criteria	Genotyping method	Quality control
				case	control			
Sharma [9]	1994	UK	European	100	73	Age and sex	PCR	No
Ueda [24]	1995	UK	European	488	188	Age and sex	PCR	Yes
Catto [25]	1996	UK	European	406	215	Age and sex	PCR	Yes
Margaglione [27]	1996	Italy	European	101	109	Sex	PCR	No
Kario [26]	1996	Japan	Asian	138	104	Age and sex	PCR	Yes
Agerholm-Larsen [28]	1997	Denmark	European	452	9038	Age	PCR	Yes
Nakata [30]	1997	Japan	Asian	55	61	Age and sex	PCR	No
Doi [29]	1997	Japan	Asian	181	271	Age and sex	PCR	Yes
Seino [32]	1998	Japan	Asian	26	28	Age and sex	PCR	No
Pfohl [31]	1998	Germany	European	91	297	**–**	PCR	Yes
Shen [33]	1998	China	Asian	44	62	Age and sex	PCR	No
Xu [34]	1998	China	Asian	65	117	Age	PCR	No
Kostulas [35]	1999	Sweden	European	96	93	Age and sex	PCR	No
Notsu [10]	1999	Japan	Asian	175	213	Sex	PCR	Yes
Zee [11]	1999	USA	European	338	338	Age	PCR	Yes
Lin [36]	2000	China	Asian	306	300	Age and sex	PCR	Yes
Wei [37]	2000	China	Asian	87	257	Age	PCR	No
Zhang [39]	2001	China	Asian	165	106	Age and sex	PCR	No
Zhang [38]	2001	China	Asian	74	72	Sex	PCR	No
Ohkubo [40]	2002	Japan	Asian	69	294	Age and sex	PCR	No
Szolnoki [41]	2003	Hungary	European	867	743	Age and sex	PCR	No
UM [42]	2003	Korea	Asian	208	636	Age and sex	PCR	No
Yuan [43]	2003	China	Asian	122	1229	Age	PCR	No
Zhang [47]	2004	Japan	Asian	151	150	Age and sex	PCR	Yes
Karagiannis [44]	2004	Greece	European	100	100	Age and sex	PCR	Yes
Wang [46]	2004	China	Asian	46	43	**–**	PCR	No
Rubattu [45]	2004	Italy	European	215	236	Age	PCR	Yes
Brenner [48]	2005	France	European	459	459	Age and sex	PCR	Yes
Pera [51]	2006	Poland	European	368	556	Age and sex	PCR	No
Dikmen [49]	2006	Turkey	European	141	50	**–**	PCR	No
Gao [50]	2006	China	Asian	100	100	Age and sex	PCR	Yes
Tuncer [52]	2006	Turkey	European	108	79	Age and sex	PCR	No
Tseng [58]	2007	China	Asian	92	780	**–**	PCR	Yes
Gormley [54]	2007	UK	European	299	600	Age and sex	PCR	No
Li [56]	2007	China	Asian	454	334	Sex	PCR	No
Lalouschek [55]	2007	Austria	European	450	817	**–**	PCR	Yes
Polupanov [57]	2007	Kirghiz	Asian	69	64	Age	PCR	No
Gawel [53]	2007	Poland	European	66	45	Age	PCR	No
Hong [59]	2008	Korea	Asian	232	225	Age and sex	PCR	No
Munshi [61]	2008	India	Asian	162	150	Age and sex	PCR	No
Mollsten [60]	2008	Sweden	European	222	542	Age and sex	PCR	Yes
Tascilar [64]	2009	Turkey	European	157	85	**–**	PCR	No
Saidi [63]	2009	Tunisia	African	228	323	Age and sex	PCR	No
Celiker [62]	2009	Turkey	European	162	107	**–**	PCR	No
Li [66]	2010	China	Asian	76	311	Age and sex	PCR	Yes
Domingues-Montanari [65]	2010	Spain	European	519	540	**–**	PCR	No
Kalita [12]	2011	India	Asian	193	188	Age and sex	PCR	Yes
Chutinet [67]	2011	Thailand	Asian	141	167	Age	PCR	No
Markoula [69]	2011	Greece	European	176	178	Age and sex	PCR	Yes
Indrajaya [68]	2011	Indonesia	Asian	30	30	Age and sex	PCR	No

### Quantitative Synthesis

Overall, the variant genotypes of *ACE* I/D polymorphism were associated with a significantly higher risk of ischemic stroke in different genetic models when all the eligible studies were pooled into the meta-analysis. As shown in Table 2, the variant heterozygote ID and homozygote DD, were associated with a significantly higher risk of ischemic stroke in a dose-response manner, compared with the wild-type homozygote II (OR = 1.16, 95% CI: 1.06–1.26 for ID and 1.54, 1.34–1.78 for DD; *P*_trend_<0.001). In addition, significant main effects were also observed in dominant model, recessive model and allele contrast model (OR = 1.29, 95% CI: 1.17–1.43, OR = 1.37, 95% CI: 1.22–1.53 and OR = 1.27, 95% CI: 1.17–1.37, respectively).

**Table 2:** Stratification analyses of the *ACE* I/D polymorphism on stroke risk.

Variables	n	ID vs II		DD vs II		DD/ID vs II (dominant)		DD vs ID/II (recessive)		D vs I allele	
		OR (95% CI)	*P* *	OR (95% CI)	*P* *	OR (95% CI)	*P* *	OR (95% CI)	*P* *	OR (95% CI)	*P* *
Total	50	1.16 (1.06–1.26)	0.017	1.54 (1.34–1.78)	<0.001	1.29 (1.17–1.43)	<0.001	1.37 (1.22–1.53)	<0.001	1.27 (1.17–1.37)	<0.001
Ethnicities											
Asian	26	1.30 (1.12–1.50)	0.010	2.20 (1.70–2.84)	<0.001	1.54 (1.30–1.82)	<0.001	1.87 (1.51–2.31)	<0.001	1.52 (1.33–1.75)	0.185
European	23	1.03 (0.94–1.12)	0.555	1.12 (1.01–1.23)	0.627	1.06 (0.98–1.15)	0.502	1.10 (1.02–1.18)	0.684	1.06 (1.01–1.12)	0.465
Source of control											
HB	28	1.14 (1.04–1.25)	0.200	1.75 (1.41–2.16)	<0.001	1.33 (1.16–1.54)	<0.001	1.53 (1.30–1.79)	<0.001	1.34 (1.20–1.50)	<0.001
PB	22	1.17 (1.01–1.35)	0.009	1.34 (1.11–1.62)	<0.001	1.24 (1.08–1.44)	0.001	1.21 (1.04–1.41)	<0.001	1.19 (1.07–1.32)	<0.001
HWE											
Yes	42	1.16 (1.06–1.28)	0.014	1.55 (1.32–1.80)	<0.001	1.29 (1.16–1.43)	<0.001	1.37 (1.22–1.54)	<0.001	1.26 (1.16–1.36)	<0.001
No	8	1.13 (0.91–1.40)	0.246	1.57 (0.99–2.48)	0.005	1.34 (0.95–1.90)	0.011	1.42 (0.97–2.10)	0.003	1.36 (1.02–1.84)	<0.001
Subtype											
SVD	17	1.16 (1.00–1.33)	0.128	1.45 (1.13–1.87)	0.011	1.25 (1.04–1.50)	0.045	1.30 (1.06–1.59)	0.006	1.20 (1.05–1.36)	0.003
LVD	17	1.09 (0.94–1.26)	0.141	1.47 (1.07–2.04)	<0.001	1.24 (0.99–1.53)	0.014	1.36 (1.04–1.76)	<0.001	1.23 (1.04–1.46)	<0.001
Gender											
Male	5	1.16 (0.76–1.79)	0.035	1.11 (0.83–1.49)	0.078	1.20 (0.78–1.85)	0.018	1.04 (0.82–1.32)	0.476	1.13 (0.87–1.45)	0.041
Female	5	1.26 (0.92–1.73)	0.090	2.16 (0.86–5.41)	0.003	1.61 (0.87–2.99)	0.014	1.46 (0.87–2.46)	0.050	1.37 (0.93–2.02)	0.008

* *P* value of Q-test for heterogeneity test.

HB: Hospital-based; PB: Population-based; SVD: small vessel disease; LVD: large vessel disease.

**Figure 2:**
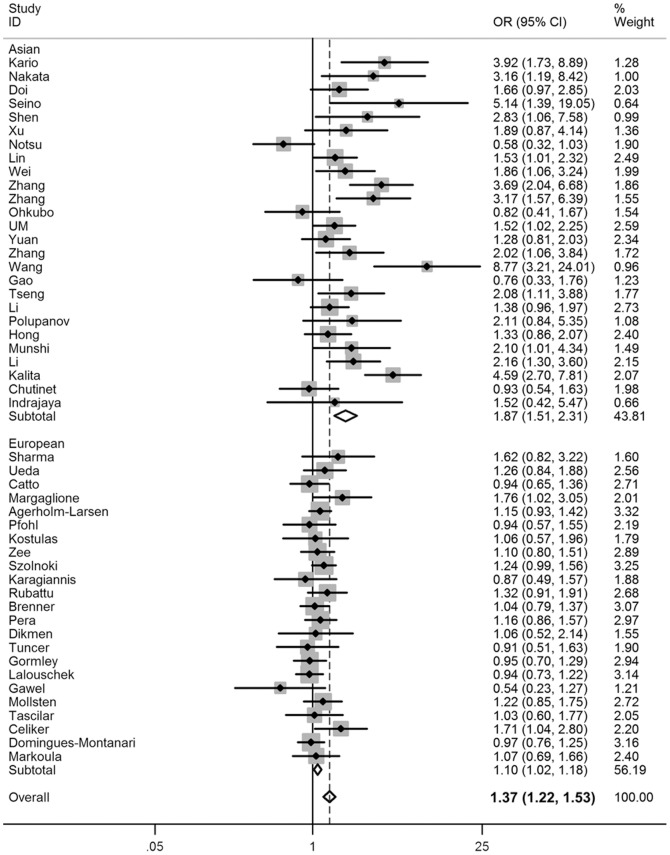
Forest plot of stroke risk associated with the *ACE* I/D polymorphism (DD vs ID/II).

In the stratified analysis by ethnicity, significantly higher risks were found in Asians (ID vs II: OR = 1.30, 95% CI = 1.12–1.50; DD vs II: OR = 2.20, 95% CI = 1.70–2.84; dominant model: OR = 1.54, 95% CI = 1.30–1.82; recessive model: OR = 1.87, 95% CI = 1.51–2.31; allele model: OR = 1.52, 95% CI = 1.33–1.75) but with borderline statistical significance in Europeans (DD vs II: OR = 1.12, 95% CI = 1.01–1.23; recessive model: OR = 1.10, 95% CI = 1.02–1.18; allele model: OR = 1.06, 95% CI = 1.01–1.12; Fig. 2). Moreover, when stratified by source of control, statistically significantly elevated risk was also observed, and this elevated risk was more pronounced among hospital-based studies (Table 2).

In the present meta-analysis, seventeen studies provided detailed genotype information according to stroke subtype. As shown in Table 2, significantly higher stroke risk was found in small vessel disease (SVD) but with borderline statistical significance in large vessel disease (LVD). However, in the subgroup analysis by gender, we did not find significant associations in any genetic models.

**Figure 3:**
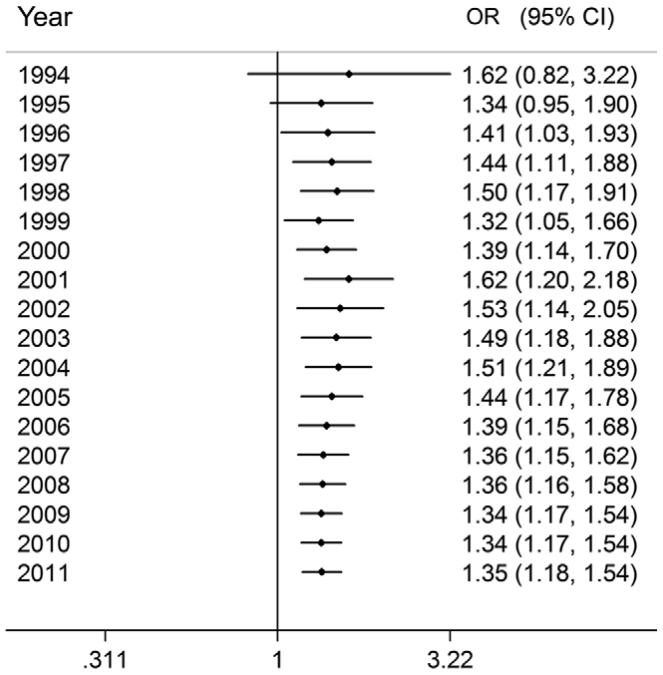
Results of the cumulative meta-analysis. The random effects pooled OR with 95% CI at the end of each information step is shown.

**Figure 4:**
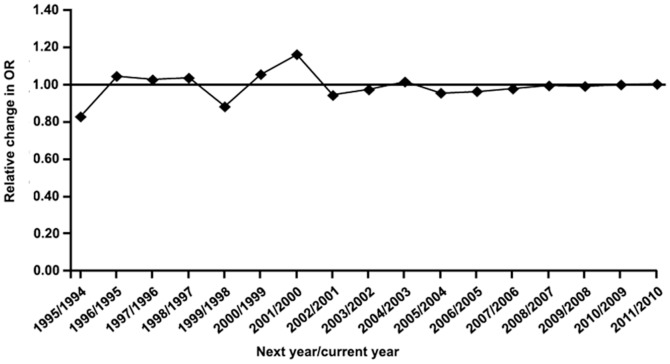
Results of the recursive cumulative meta-analysis. The relative change in random effects pooled OR in each information step (OR in the next year/OR in the current year) for the recessive model is shown.

The cumulative meta-analysis for the recessive model showed a trend of association as published data information accumulated (Fig. 3). In recursive cumulative meta-analysis, the relative change in the random effects ORs fluctuated around 1.00 until 2008 and then stabilized, indicating that there is sufficient evidence for investigating the association (Fig. 4).

### Test of Heterogeneity

Obvious heterogeneity between studies was observed in overall and subgroup analyses (*P*<0.05, *I* ^2^>30%, Table 2). Then, we assessed the source of heterogeneity for recessive model comparison by ethnicity, sample size (subjects >800), and source of controls. As a result, ethnicity (χ^2^ = 33.13, df = 2, *P*<0.001) and source of controls (χ^2^ = 13.99, df = 1, *P*<0.001), but not the sample size (χ^2^ = 3.13, df = 1, *P* = 0.077), was found to contribute to substantial heterogeneity.

### Sensitivity Analyses

Sensitivity analyses indicated that four independent studies [10], [12], [39], [46] were the main origin of the heterogeneity in Asians. The heterogeneity was effectively lowered or removed after exclusion of these four studies (DD vs ID/II: *P*_heterogeneity_ = 0.081). Although the genotype distributions in eight of the included studies did not follow HWE, the corresponding pooled ORs were not materially altered with or without including these studies (Table 2). In further sensitivity analysis in which 1 study at a time was removed and the rest analyzed, the pooled ORs ranged from 1.32 to 1.39, indicating that the pooled estimate was robust and not influenced by a single study.

### Publication Bias

Visual inspection of the Begg's funnel plot showed some asymmetry. Then, the Egger's test was used to provide statistical evidence of funnel plot asymmetry. As expected, the results indicated an obvious evidence of publication bias (t = 3.70, *P* = 0.001 for DD vs ID/II). Random-effects OR corrected for publication bias using the trim and fill method was 1.19 (95% CI, 1.05–1.35) for all studies combined. Correction for potential publication bias therefore did not materially alter the combined risk estimate.

## Discussion

The present meta-analysis, including 10 070 cases and 22 103 controls from 50 published studies, explored the association between the *ACE* I/D polymorphism and stroke risk. We found that the variant genotypes of this polymorphism were associated with significant increase in overall stroke risk.

ACE activates angiotensin I and inactivates bradykinin, resulting in decreased tissue perfusion, vascular smooth muscle cell growth [70], and stimulation of plasminogen-activator inhibitor type I [71]. Moreover, plasma ACE concentration is an important factor in cardiovascular and cerebrovascular risk profiling, since chronic exposure to high levels of plasma ACE may result in vascular wall thickness and stiffness [72]. Thus, the *ACE* gene is a good candidate gene for ischemic stroke. Early studies demonstrated a strong correlation between the D allele and levels of circulating, intracellular, and tissue activity of ACE [8], [73]. Since both alleles have codominant effects on ACE levels, individuals who are homozygous for the D allele have the highest levels of the enzyme, those homozygous for the I allele have the lowest, and heterozygous individuals have an intermediate level. Given the important roles of ACE in the pathogenesis of cerebrovascular disease, it is biologically plausible that *ACE* polymorphism may modulate the risk of ischemic stroke. In present meta-analysis, we found that variant genotypes of *ACE* I/D polymorphism were associated with higher stroke risk, which was consistent with experimental findings.

Significant associations were found in Asians but with borderline statistical significance in Europeans, suggesting a possible role of ethnic differences in genetic backgrounds and the environment they lived in. The impact of this polymorphism may be masked by the presence of other as-yet unidentified causal genes involved in stroke development in Europeans. Other factors such as selection bias, different matching criteria may also play a role. The above differences may lead to the inconsistent results. In addition, there is only one reported study using African population for this polymorphism. So it is also likely that the observed ethnic differences may be due to chance because studies with small sample size may have low statistical power to detect a slight effect. Thus, additional studies are warranted to further validate ethnic difference in the effect of this polymorphism on stroke risk, especially in Africans.

In the subgroup analysis by subtype, significantly higher stroke risk was observed in SVD but with borderline statistical significance in LVD. Stroke is a heterogenous disease and it is possible that *ACE* I/D polymorphism may play different roles in the differing subtypes of strokes. Our findings indicate that genetic risk factors for different subtypes are likely different, supporting the view that they are pathologically distinct entities, with SVD having a greater genetic liability compared to LVD.

No significant association between variant genotypes and stroke risk was observed when the included studies were stratified by gender. The null result may be due to limited number of studies with available data, which had insufficient statistical power to detect a slight effect or may have generated a fluctuated risk estimate.

Our findings confirmed results from previous meta-analyses. With the accumulative evidence, we were able to enhance the precision of the risk estimates and perform subgroup analyses to explore sources of heterogeneity, thereby increasing the clinical relevance of our findings. However, some limitations of this meta-analysis should be addressed. First, lacking of the original data of the reviewed studies limited our further evaluation of potential interactions, because the gene-gene interaction and gene-environment interaction may modulate stroke risk. Second, a potential publication bias may exist, as shown by the funnel plot and the Egger's test. Nevertheless, correction for this bias using the trim and fill method did not materially alter the combined risk estimate.

In conclusion, this meta-analysis provided evidence of the association between the *ACE* I/D polymorphism and stroke risk, supporting the hypothesis that the *ACE* I/D polymorphism may be a low-penetrance susceptibility marker of stroke. However, additional large studies are warranted to validate our findings. Future studies should use standardized genotyping methods and homogeneous patients and well-matched controls and include multi-ethnic populations. Furthermore, detailed gene-gene interaction and gene-environment interaction should also be considered in future studies, which should lead to better understanding of the association between the *ACE* I/D polymorphism and stroke risk.
